# Advancing the Progress of Mentoring for Diversity in AIDS Research: Warming the Mentoring Climate

**DOI:** 10.1007/s10461-016-1490-y

**Published:** 2016-07-23

**Authors:** Hannah A. Valantine

**Affiliations:** National Institutes of Health, Building 1 Room 316, 1 Center Drive, Bethesda, MD 20814 USA

There is no doubt that in 2016, after many years of attempts to diversify the U.S. biomedical workforce, we are not yet there. Currently, the nation’s biomedical workforce of researchers, physicians, and public health professionals does not mirror our nation’s demographic diversity. A workforce lacking in diversity is especially troubling for disease areas such as HIV/AIDS that disproportionately affect underserved populations. For example, according to the U.S. Centers for Disease Control and Prevention, the greatest number of new HIV infections among gay, bisexual, and other men who have sex with men (MSM) occurred in young black/African American MSM 13–24 years old. By race, blacks/African Americans face the most severe burden of HIV.

Thus, we have a lot to gain by whatever measures contribute to increasing diversity in the HIV/AIDS workforce. In so doing, we can broaden creative thinking by inviting diverse points of view; we can adjust the research agenda to be more scientifically and culturally relevant to those affected by HIV/AIDS; and we can enhance efforts to improve clinical research participation by at-risk individuals. Collectively, these actions should contribute to reducing disease burden.

A host of programs, experiences, and research has taught us that pipeline solutions to increasing diversity are necessary, but far from sufficient. Juncture points along the early-career trajectory (late doctoral, post-doctoral entry/exit, faculty entry) should be targeted to buffer against breaks at these critical career transitions in which personnel, geographic, and cultural influences may change dramatically and prompt highly trained scientists to leave science. The transition from trainee to career independence appears most vulnerable: recent studies on career choices being made by graduate students reveal a significant attrition by all groups away from biomedical research careers, an observation that disproportionally affects women and individuals from underrepresented groups (URG) [1]. Faculty relationships, mentorship, self-efficacy, research training experiences, and objective performance measures traditionally associated with scholarly productivity are important variables that affect undergraduate student choices for research careers [2]. However, upon completion of Ph.Ds. and postdoctoral fellowships, central drivers for pursuing a faculty career—across racial, ethnic, and gender backgrounds—are personal values and perceptions of structural dynamics [3]. Women and URG scientists with high interest in obtaining faculty positions are motivated by the opportunity to engage according to externally focused values they deem important. For example, these include the ability to conduct research applicable to pertinent health problems in communities; having meaningful effects on students; and being role models. In contrast, for scientists from racial/ethnic *majority* backgrounds, academic freedom to pursue research topics of interest is an important driver of career choice. For scientists choosing careers *outside academia*, the principal value, articulated across social identity, is having a higher level of work applicability than is perceived to be attainable in a research-university setting.

In addition to personal values, structural dynamics often discourage interest in faculty careers; these include the academic job market, availability of grant funding, and postdoctoral pay. Of note, structural dynamics; e.g., career–life balance issues and an unsupportive institutional climate experienced during gradate training, differ across social identities. Recent findings underscore the complexity of career choice and the need for further study to understand the effects of social identity and its impact on scientific workforce diversity. The available data offer some guidance to interventions targeted at structural dynamics and barriers to career-juncture transition. For example, a multifaceted intervention that provided structured faculty career-development opportunities and programs to minimize social isolation increased the proportion of women at every rank, including full professor [4]. Similarly, interventions that help faculty address career/life challenges combined with approaches targeted at institutional and professional cultures aimed at destigmatizing use of career-flexibility options are likely to enhance retention [5, 6]. In addition, interventions to ameliorate the negative effects of unconscious bias will likely also enhance the interest and diversity of trainees pursuing academic careers [7].

Many factors inform scientific workforce disparities, and sociocultural factors that affect recruiting and retention are significant. These include, but are not limited to, unconscious bias, stereotype threat, belonging, and cultural climate. Although an under-diverse workforce has traditionally been viewed as a problem—and in the representation sense, it is—I propose that we take a fresh look at diversity. Let’s consider achieving diversity, in its many forms, as an extraordinary opportunity for twenty-first century biomedicine—in an era of HIV/AIDS in which major progress has already been made and we are on the cusp of remarkable advances in prevention, targeted treatment, and even sustained remission. Arguably the most important, yet least understood and quantifiable, aspect of recruiting and retaining diverse minds and approaches is mentorship.

Effective mentoring is a crucial element to creating and retaining diversity. This special supplement issue of *AIDS and Behavior* highlights central issues in mentoring and diversity scholarship and application. It presents a contextual view of current research programs that inform development of a robust and diverse HIV/AIDS workforce that spans basic research to public health. Of particular interest is a spotlight on those sociocultural factors that influence an individual’s access to, and mastery of, both the hard and soft skills required for a successful biomedical career. These individual and institutional factors include unconscious bias, stereotype threat, benevolent sexism/racism, and identity.

Despite its centrality in high-quality research, mentoring is sometimes a fuzzy concept for many people. The term still commonly implies a trainer-trainee dyad, but it should be construed as a much larger notion of providing support to scientists throughout the research continuum: not just at the beginning, and not just in the context of a single lab or project. Emerging concepts along these lines include coaching, promoting an inclusive climate for learning and leading, sponsorship for advancing important career opportunities, and encouragement of multidisciplinary mentoring teams. Diverse mentoring models have been approached successfully, including distance mentoring, peer mentoring, functional skill mentoring, and others. Mentoring-in-place is another exciting concept that aims to embed mentoring within ongoing research projects and networks. Many of these mentoring concepts have direct relevance to the complex intersection of biology, behavior, and culture that define the HIV/AIDS research landscape and patient experiences.

It is possible to “measure” mentoring, in the sense that as a research community we can develop recognizable hallmarks of success and trace the paths that lead individuals to attaining necessary skills and opportunities. Researchers are studying mentoring in the context of institutional practices—essential work that should be expanded. One recent study, an NIH-funded randomized controlled trial (RCT) conducted across several academic medical centers, showed that systematic, formalized competency-based research mentor-training improved not only self-reported mentor skills but also mentoring behavior [8]. This work built upon earlier NIH-funded research that established the mentoring competency assessment (MCA), a 26-item skills inventory that enables mentors and mentees to assess various competencies [9]. Another NIH-funded RCT involving URG individuals showed positive, albeit short-term, benefit from mentor training on mentees’ psychological need satisfaction with mentors [10].

We know that the research and training infrastructure that is available at research-intensive institutions is of value, but what specific elements of these experiences actually define biomedical interest and career success for individual students? We also know the value of mentoring, in general, but what, precisely, constitutes “effective research mentoring?” Moreover, how does culture influence mentoring success? These are many questions begging for rigorous study.

To that end, in October 2014, NIH announced the first awardees of a five-year, $250 million (total) NIH Diversity Consortium, designed to develop approaches that engage trainees, including those from underserved backgrounds, and prepare them to thrive in NIH-funded research careers. Consisting of three integrated elements: the Building Infrastructure Leading to Diversity (BUILD) program (ten institutions), the National Research Mentoring Network (NRMN), and the Coordinating and Evaluation Center (CEC), this investment creates a consortium focused on scientifically driven approaches to enhancing workforce diversity. Through this NIH Common Fund-supported initiative, we are looking behind the curtain at programs and paradigms to understand (and replicate) effective strategies for student engagement, research training, mentoring, faculty development, and infrastructure development. Many other efforts at other agencies, academic institutions, and organizations have placed a much-needed emphasis on mentoring writ large. Several of these have been described by the NIH-funded understanding interventions project [11].

The NIH-funded NRMN is developing best practices for mentoring, providing training opportunities for mentors, and providing networking and professional opportunities for mentees. In addition to what we are learning from the vibrant community of scholars studying mentoring, it is important to consider mentoring in the context of modern biomedicine. Academic institutions provide the workplaces wherein a large proportion of the future research workforce is trained and launched into independent careers. Unfortunately, however, academic workplace cultures, designed for workers of the past century, do not meet the needs of twenty-first century workers who are typically part of dual-career families that need much greater flexibility to enable life-work integration. This is an issue for both women and men. Preparing tomorrow’s biomedical researchers and leaders must include strategies to foster a supportive culture for life-work integration. Mentors can play a key role in this task.

Going forward, a challenge before us is to take the data we have gathered and learn how to apply it contextually toward the scholarship of mentoring and to improving on-the-ground experiences. To this end, it is useful to consider HIV/AIDS research mentoring and capacity-building experiences from the perspective of minority mentees, a topic discussed herein. Institutional commitment to diversity, and the role (and reward) of mentoring is paramount. Without strong leadership from government and academic circles, we will continue to have idiosyncratic success in ensuring that mentoring is valued as a key determinant of research excellence. Authors featured in this supplement have visited this central issue.

I will end at the beginning, by stressing that our scientific workforce is not as diverse as it could be, but that opportunities abound to mount this as a scientific challenge. Imagine the possibilities of thinking differently, of approaching new angles to health problems, of changing the face of clinical research. To reap the opportunities of a diverse workforce, especially in the field of HIV/AIDS, will require a warming climate offered by mentoring. In so doing, we will serve the needs of the heart of biomedicine: the patient.
